# The Effect of Mushroom Extracts on Human Platelet and Blood Coagulation: In vitro Screening of Eight Edible Species

**DOI:** 10.3390/nu11123040

**Published:** 2019-12-12

**Authors:** Barbara Poniedziałek, Marek Siwulski, Adrian Wiater, Iwona Komaniecka, Anna Komosa, Monika Gąsecka, Zuzanna Magdziak, Mirosław Mleczek, Przemysław Niedzielski, Jędrzej Proch, Mariola Ropacka-Lesiak, Maciej Lesiak, Eliana Henao, Piotr Rzymski

**Affiliations:** 1 Department of Environmental Medicine, Poznan University of Medical Sciences, 60-806 Poznan, Poland; 2 Department of Vegetable Crops, Poznan University of Life Sciences, 60-594 Poznan, Poland; marek.siwulski@up.poznan.pl; 3 Department of Industrial Microbiology, Maria Curie-Sklodowska University in Lublin, 20-033 Lublin, Poland; adrianw2@poczta.umcs.lublin.pl; 4 Department of Genetics and Microbiology, Institute of Biological Sciences, Maria Curie-Sklodowska University, 20-033 Lublin, Poland; ikoma@hektor.umcs.lublin.pl; 5 1st Department of Cardiology, Poznan University of Medical Sciences, 61-848 Poznan, Poland; komosa.ania@gmail.com (A.K.); maciej.lesiak@ump.edu.pl (M.L.); 6 Department of Chemistry, Poznan University of Life Sciences, 60-625 Poznań, Poland; buba@up.poznan.pl (M.G.); zuzanna.magdziak@up.poznan.pl (Z.M.); mirekmm@up.poznan.pl (M.M.); 7 Department of Analytical Chemistry, Faculty of Chemistry, Adam Mickiewicz University, 61-614 Poznań, Poland; pnied@amu.edu.pl (P.N.); jed.proch@gmail.com (J.P.); 8 Department of Perinatology and Gynecology, Poznan University of Medical Sciences, 60-535 Poznan, Poland; mariolaropacka@poczta.onet.pl; 9 Department of Biology, Universidad del Valle, 100-00 Cali, Colombia; eliana.henao@correounivalle.edu.co

**Keywords:** mushrooms, platelet, natural antiplatelet agents, thrombosis, cardiovascular disease

## Abstract

Cardiovascular diseases remain the leading global cause of mortality indicating the need to identify all possible factors reducing primary and secondary risk. This study screened the in vitro antiplatelet and anticoagulant activities of hot water extracts of eight edible mushroom species (*Agaricus bisporus*, *Auricularia auricularia-judae*, *Coprinus* *comatus*, *Ganoderma* *lucidum*, *Hericium erinaceus*, *Lentinula* *edodes*, *Pleurotus* *eryngii*, and *Pleurotus* *ostreatus*) increasingly cultivated for human consumption, and compared them to those evoked by acetylsalicylic acid (ASA). The antioxidant capacity and concentration of polysaccharides, phenolic compounds, organic acids, ergosterol, macro elements, and trace elements were also characterized. The most promising antiplatelet effect was exhibited by *A*. *auricularia*-*judae* and *P*. *eryngii* extracts as demonstrated by the highest rate of inhibition of adenosine-5′-diphosphate (ADP)-induced and arachidonic acid (AA)-induced aggregation. The response to both extracts exceeded the one evoked by 140 µmol/L of ASA in the ADP test and was comparable to it in the case of the AA test. Such a dual effect was also observed for *G*. *lucidum* extract, even though it was proven to be cytotoxic in platelets and leukocytes. The extract of *P*. *ostreatus* revealed an additive effect on AA-induced platelet aggregation. None of the mushroom extracts altered the monitored coagulation parameters (prothrombin time, prothrombin ratio, and International Normalized Ratio). The effect of mushroom extracts on platelet function was positively related to their antioxidative properties and concentration of polysaccharides and ergosterol, and inversely related to zinc concentration. The study suggests that selected mushrooms may exert favorable antiplatelet effects, highlighting the need for further experimental and clinical research in this regard.

## 1. Introduction

Cardiovascular disease (CVD) is the leading cause of morbidity and mortality in the world, regardless of race, ethnicity, or gender. In Europe, CVD is responsible for approximately 3.9 million deaths annually and generates enormous medical costs [1,2]. It is of particular and pressing interest to implement effective primary and secondary prevention strategies that include smoking cessation, increase in physical activity, and weight and diet optimization [3,4,5]. It has also been established that a reduction in platelet aggregation would lead to a significant decrease in the risk of myocardial infarction and the total number of cardiovascular events [6,7]. In patients with diagnosed CVD, antiplatelet therapy that involves pharmaceuticals, such as acetylsalicylic acid (ASA), has been proven to reduce the risk of major cardiovascular events. However, the benefits of ASA in primary prevention have been controversial [8]. According to the 2016 European Guidelines on cardiovascular prevention in clinical practice, ASA prophylaxis is not recommended for subjects who do not suffer from CVD because of an increased risk of major bleeding [9]. It has also been demonstrated that time of administration strongly affects the outcome of ASA on platelet function [10]. This creates a necessity to seek alternative and safe dietary antiplatelet agents.

Recently, an antiplatelet effect of tomato extract, mostly attributed to the presence of phenolic compounds, has been well documented in in vitro experimental studies. Its partial benefits in the primary prevention of CVD have been reported for obese subjects [11,12]. A growing interest has also been expressed in the use of mushrooms as functional foods [13,14], and, in recent decades, certain mushroom species have been experimentally found to exert antiplatelet effects through different pathways. For example, an extract of *Hericium erinaceus* was found to selectively impede collagen signaling from GPIIb/Ia [15], which is an extract of *Phellinus baummii* suppressed collagen-5′diphosphateinduced, thrombin-5′diphosphateinduced, and adenosine-5′diphosphateinduced aggregation [16]. A methanol extract of *Pleurotus florida* inhibited adenosine-5′diphosphate-induced aggregation in isolated human platelets [17] while a dry extract of *Stereum hirsutum* diminished platelet activation by blocking the thrombin active site [18]. Recently, aqueous extracts of *Pleurotus* mushrooms have been demonstrated to successfully prevent an increase in platelet’s concentration of reactive oxygen species (ROS), which are known to mediate platelet activation via different pathways [19,20]. Altogether, these findings highlight the need to further explore and compare the potential antiplatelet activities exerted by different mushrooms. Edible species are of particular interest since they are increasingly popular as foodstuffs in different world locations due to their nutritional value and potential medicinal use, which is extensively investigated over recent decades [14,21].

The present study aimed to assess and compare the effects of hot water extracts obtained from eight edible mushroom species cultivated for the purpose of this research, which included *Agaricus bisporus* (J.E. Lange) Imbach, *Auricularia auricularia-judae* (Bull.) J. Schröt, *Coprinus comatus* (O.F. Müll.) Pers., *Ganoderma lucid*um (Curtis) P. Karst., *Hericium erinaceus* (Bull.) Pers., *Lentinula edodes* (Berk.) Pegler, *Pleurotus eryngii* (DC.) Quél., and *Pleurotus ostreatus* (Jacq.) P. Kumm. on human platelet function and blood coagulation parameters. The hot water extracts were specifically utilized since they more closely reflect the situation of mushroom consumption compared to organic-solvent extracts. A number of in vitro assays have been employed to (i) test the effect of mushroom extracts on platelet aggregation induced by adenosine-5′diphosphate (ADP) and arachidonic acid (AA), (ii) evaluate the effect of mushroom extracts on prothrombin time and the international normalized ratio, and (iii) assess the safety of extracts by investigating their cytotoxicity to human platelets, leukocytes, and erythrocytes. The observed effects were compared to those evoked by ASA, which is a non-selective and irreversible inhibitor of platelet cyclooxygenase 1 (COX-1) involved in arachidonic-acid transformation [22], and an inhibitor of the lectin-like oxidized low-density lipoprotein receptor-1 (LOX-1) whose expression on platelets increased upon their exposure to ADP [23]. All extracts were also characterized by several parameters including concentration of polysaccharides, phenolic compounds, organic acids, ergosterol, trace elements, and total antioxidant capacity.

## 2. Materials and Methods

### 2.1. Mushroom Cultivation

The substrate for five species of mushrooms i.e., *P. eryngii*, *G. lucidum*, *H. erinaceus*, *L. edodes*, and *A. auricula-judae* was prepared from a mixture of beech and oak sawdust (3:1 vol.), which is additionally supplemented with wheat bran in the amount of 25%, corn flour of 5%, and gypsum of 1% in relation to the substrate dry matter. The mixture was moistened to a moisture content of 65% and sterilized at 121 °C for 1 h. The substrate for *P. ostreatus* was prepared from wheat straw chaff (4–5 cm long). The straw was moistened to a moisture content of 65% and pasteurized in 60 °C for 24 h. The substrate for *A. bisporus* and *C. comatus* was prepared from wheat straw, chicken manure, and gypsum (1000 kg, 900 kg, and 85 kg, respectively) using the conventional method characteristic for phase II (fermentation and pasteurization). The details of substrate preparation have been given in previous works [24,25,26]. In all cases, the fruiting bodies from the first flush of cropping were used to prepare extracts.

### 2.2. Extract Preparation

Freshly collected fruiting bodies of each species were immediately homogenized after harvesting using a cutting mill (Cloer, Arnsberg, Germany), and 50 mg of the obtained material was then extracted in distilled water at a ratio of 1:10 (*w*/*v*) at 80 °C for 120 min. The samples were then cooled to room temperature and centrifuged. The supernatants were sterilized by filtration on 0.22-μm syringeless filter devices (Carl Roth, Karlsruhe, Germany). The extracts were kept in small aliquots at −80 °C and thawed to room temperature before each use.

### 2.3. In vitro Studies

#### 2.3.1. Blood Collection

All blood samples used in the present study were purchased from the Regional Centre of Blood and Blood Treatment in Poznan, Poland, according to accepted safeguard standards and legal requirements in Poland. The inclusion criteria were as follows: normal BMI (18.5–24.9 kg/m^2^), age 25–35 years old, non-smoking, normal hematological parameters (percentage of lymphocytes, monocytes, neutrophils, basophils, eosinophils, platelet and red blood cell count, hematocrit value, and hemoglobin concentration), no use of ASA or clopidogrel at least for the last 14 days. For the aggregation assay, whole blood samples were collected in S-Monovette^®^ (Sarstedt, Nümbrecht, Germany) coated with hirudin, which is a desired anticoagulant for impedance aggregometry [27]. For coagulation and viability assays, venous blood samples were collected into tubes containing 3.2% sodium citrate as an anticoagulant (S-Monovette^®^ 9NC, Sarstedt, Nümbrecht, Germany). The ratio of blood to anticoagulant was no less than 10:1. To conduct the cytotoxicity assay on platelet-rich plasma (PRP) and leukocytes, blood was collected into tubes containing acid citrate dextrose and lithium heparin, respectively. To conduct the erythrocyte sedimentation rate assay, blood samples were collected directly into Microvette CB200 capillary tubes (Sarstedt, Nümbrecht, Germany) containing 3.2% sodium citrate as an anticoagulant (4:1). For each assay, venous blood samples from five donors were obtained. This sample size was selected based on the number of independent replicates employed by other in vitro studies assessing the antiplatelet and anticoagulation function of different agents [28,29,30].

#### 2.3.2. Aggregation Assay

A Multiplate^®^ Platelet Function Testing 5.0 Analyzer (Roche, Rotkreuz, Switzerland) based on multiple electrode aggregometry was used to measure an agonist-induced platelet aggregation. Whole blood samples (350 µL) were then incubated with 35 µL of mushroom extract, ASA (140 µmol/L), or phosphate-buffered saline (PBS; control) for 10 min at 37 °C. Following the standardized setup of the Multiplate^®^ 5.0 Analyzer (Roche Diagnostics, Mannheim, Germany), test cuvettes were loaded with 300 μL of NaCl. Next, 300 μL of whole blood was electronically pipetted into each cuvette followed by a three-minute incubation while the sample was stirred automatically. After the incubation, 20 μL of the agonist was added to each respective cuvette followed by a six-minute incubation period. At the end of six minutes, platelet aggregation was measured in duplicate as electrical impedance. Two test assays were performed: an ADPtest (platelet aggregation in response to adenosine-5′-diphosphate) and an ASPItest (aggregation in response to AA). The results were expressed as U (1U = 10AU* min). The normal reference range in the ADPtest and ASPItest is 53–122 and 75–136 AU, respectively. Blood samples of patients whose unexposed platelets did not fall within these ranges were excluded from the study.

#### 2.3.3. Coagulation Assay

The effects of extracts on the blood coagulation level were assessed with the prothrombin time measurement and calculation of the international normalized ratio (INR). This assay was specifically selected since it was shown to be a more reliable indicator of altered coagulation factor levels if compared to the activated partial thromboplastin time (APTT) assay, which is prone to false positive results under certain conditions, e.g., elevated factor VIII [31]. Plasma was obtained by centrifugation at 1500 × g at 21 °C for 15 min, pipetted, divided into 350 µL aliquots, and incubated with 35 µL of mushroom extract, ASA (140 µmol/L), or PBS (control) for 10 min at 37 °C. The prothrombin time was measured immediately in a certified hematological laboratory by the optical nephelometric method using a K-3002 Optic coagulometer (Kselmed, Grudziądz, Poland). INR values were calculated using the formula below.

(1) $$\mathrm{INR}={\left(\frac{\mathrm{prothrombin}\;\mathrm{time}\;\mathrm{for}\;\mathrm{sample}}{\mathrm{prothrombin}\;\mathrm{time}\;\mathrm{for}\;\mathrm{laboratory}\;\mathrm{reference}\;\mathrm{plasma}}\right)}^{\mathrm{ISI}}$$

where ISI stands for international sensitivity index for thromboplastin. The normative laboratory range for prothrombin time and INR was 12–16 s and 0.9–1.2 s, respectively.

#### 2.3.4. Cytotoxicity Assay

To evaluate whether mushroom extracts may alter human cell viability, which would exclude them from any practical use, a lactate dehydrogenase (LDH) assay on platelet-rich plasma and isolated leukocytes was performed. LDH is a widely used cytotoxicity marker since cells undergoing death lose their membrane permeability and release LDH rapidly to the extracellular environment in which it remains relatively stable [32]. PRP was obtained by centrifugation at 200 g for 12 min, carefully removed, and divided into 10 aliquots. Each was exposed to mushroom extract (1:10 *v*/*v*), ASA (140 µmol/L), or PBS (control) for 10 min. Leukocytes were isolated from venous blood using a one-step density-gradient centrifugation on Gradisol G of a specific gravity of 1.115 g mL^−1^ (Polfa, Warszawa, Poland) at 400 g at room temperature for 30 min. The residual erythrocytes were removed from the cell population by hypotonic lysis. The purity of the leukocytes (>95%) was verified by counting under a light microscope after May-Grünwald-Giemsa staining. The exposure was carried out in a similar fashion as in the case of PRP. Following the incubation, samples of PRP and leukocytes were centrifuged at 200 g for 10 min to pellet cells, and the activity of total LDH in the supernatants was measured spectrophotometrically with a Lactate Dehydrogenase Activity Assay Kit (Sigma-Aldrich, St. Louis, MO, USA), according to the manufacturer’s instructions and with a Synergy HTX multi-mode plate reader (BioTek Instruments, Winooski, VT, USA). Low control was constituted of untreated PRP or leukocytes. High control was constituted of PRP or cells treated with Tritox X-100. Cytotoxicity was calculated from absorbance values according to the following formula.

(2) $$\%\;\mathrm{Cytotoxicity}=\frac{\mathrm{exposed}\;\mathrm{sample}\;-\;\mathrm{low}\;\mathrm{control}}{\mathrm{high}\;\mathrm{control}-\mathrm{low}\;\mathrm{control}}\;x\;100$$

Considering the short exposure time, values exceeding 5% were considered to indicate cytotoxicity in human platelets and leukocytes.

#### 2.3.5. Erythrocyte Sedimentation Rate Assay

To assess whether mushroom extracts may affect the erythrocyte sedimentation rate (ESR), 20 μL of each extract, ASA (140 µmol/L), or PBS (control) was added to a tube containing 200 µL of collected blood. Tubes were inverted eight times to mix and insert into the dedicated ESR rack (Sarstedt, Nümbrecht, Germany) for 1 h. ESR was given as millimeters of sedimented red blood cells. The value < 20 mm/h was considered as a reference range.

### 2.4. Extract Characterization

#### 2.4.1. Total Antioxidant Capacity

The total antioxidant capacity of the mushroom extracts was determined by measuring their ability to scavenge the radical cation of 2,2′-azino-bis(3-ethylbenzothiazoline 6-sulphonate) (ABTS) [33]. For this purpose, an extract sample was mixed with 150 µM ABTS and 2.5 µM myoglobin. The reaction was initiated with 75 µM of hydrogen peroxide. Following the 5 min of incubation at 21 °C, the absorbance was read at 734 nm, and values were related to a calibration curve (r^2^ = 0.98). It was prepared using the 0.5–2.0 mM of water-soluble analogue of vitamin E (6-hydroxy-2,5,7,8-tetramethylchroman-2-carboxylic acid, Trolox) (Sigma-Aldrich, St. Louis, MO, USA), and presented as mM of Trolox equivalents. Three technical replicates were performed for each sample.

#### 2.4.2. DPPH Scavenging Activity

The scavenging of the DPPH radicals was performed as proposed by Dong et al. [34]. One mL of each extract was mixed with a MeOH solution of 2,2-diphenyl-1-picrylhydrazyl (DPPH) free radical (2.7 mL of 6 µmol/L), shaken, and incubated for 1 h in the dark. The reduction of the DPPH in each sample was measured at 517 nm, and the scavenging activity was calculated according to the formula: [(Ac − A)/Ac] × 100, where Ac is the absorbance of the control and A is the absorbance of the samples [35].

#### 2.4.3. Total Phenolic Content

The total phenolic content (TP) was determined by the Folin–Ciocalteu assay [36]. The absorbance of 1 mL extract mixed with 1 mL of Folin–Ciocalteu reagent (diluted with H20, 1:1, *v*/*v*) and 3 mL of 20% Na_2_CO_3_ was measured at 765 nm after 30 min incubation in darkness at room temperature.

#### 2.4.4. Phenolic Compounds and Organic Acid Concentration

Apart from the total phenolic compound, the presence of the following phenolic compounds was determined: p-coumaric, gallic, protocatechuic, benzoic, caffeic, chlorogenic, 2,5-dihydroxybenzoic, ferulic, 4-hydroxybenzoic, salicylic, sinapic, syringic, vanillic, and trans-cinnamic acids as well as catechin, kaempferol, luteolin, rutin, quercetin, and vitexin. The organic acid concentration in mushroom extracts was also determined: acetic, citric, formic, fumaric, lactic, maleic, malic, malonic, oxalic, succinic, and quinic acids.

For the purpose of this analysis, the mushroom extracts were dissolved in 1 mL of 80% MeOH and an Acquity UPLC H-Class System equipped with a PDA eλ Detector (Waters Corp., Milford, MA, USA) was employed. A gradient elution of solvent A (water with 0.1% formic acid) and solvent B (acetonitrile with 0.1% formic acid) was used on an Acquity UPLC BEH C18 column (2.1 mm × 150 mm, 1.7 µm, Waters Corp., Milford, MA, USA). The following program was applied: flow 0.4 mL/min—5% B (2 min), 5–16% B (5 min), 16% B (3 min), 16–20% B (7 min), 20–28% B (11 min) flow 0.45 mL/min—28% (1 min), 28–60% B (3 min) flow 5.0 mL/min—60–95% B (1 min), 65% B (1 min), and 95–5% B (0.1 min) flow 0.4 mL/min—5% B (1.9 min). The injection volume was 5 µL. Compounds were identified by comparing the retention time of the analyzed peaks with the retention time of standards and/or by adding an external standard. All samples were repeated three times and quantified by comparing the area of their peaks recorded in preferable wavelengths at 280 and 320 nm with calibration curves obtained from commercial standards of each compound obtained from Sigma-Aldrich (St. Louis, MO, USA) [37,38].

#### 2.4.5. Separation and Compositional Analysis of Polysaccharides

The extracts of fruiting bodies were centrifuged (15 min, 12,000 rpm) in a high speed centrifuge (model 6K15, Sigma, Nümbrecht, Germany) to separate the supernatant and the residue. The supernatants were precipitated with four volumes of ethanol (96%, *v*/*v*), and then kept at 4 °C overnight in a refrigerator to precipitate polysaccharides. The precipitates formed in the solution were collected, re-dissolved in Milli-Q water, and then centrifuged (15 min, 12,000 rpm). The supernatants were further frozen at −40 °C and lyophilised in a vacuum freeze dryer (model Freezone 6, Labconco, Kansas City, MO, USA). The crude polysaccharides were obtained.

Mono-sugars were liberated from the polysaccharides by hydrolysis using 2 M trifluoroacetic acid (100 °C, 4 h), then converted into alditol acetates by reducing NaBD4 and peracetylation, which was described elsewhere [39]. The quantitative analysis was performed using inositol as an internal standard, and, additionally, the amount of each sugar component was calculated as the percentage of crude polysaccharide extract.

The linkages position in polysaccharides was established by methylation, according to the method of Ciukanu and Kerek [40]. Permethylated products were extracted into chloroform, acid hydrolysed (2 M TFA, 100 °C, 4 h), reduced with NaBD4, and peracetylated. Obtained sugar derivatives (alditol acetates, peracetylated butyl glycosides, and partly methylated alditol acetates) were analyzed by gas chromatography–mass spectrometry (GC-MS). GC-MS, carried out on an Agilent Technologies gas chromatograph (7890A) connected to a mass selective detector (5975C inert XL EI/CI MSD), and equipped with a capillary column HP-5MS (30 m × 0.25 mm). The carrier gas was helium, with a flow rate of 1 mL/min. The temperature program was as follows: 150 °C for 5 min, raised to 310 °C at 5 °C/min, and kept for 10 min.

#### 2.4.6. Organic Acid Concentration

The concentration of the organic acids in mushroom extracts was determined using Waters Alliance 2695 Chromatograph with a Waters 2996 Photodiode Array Detector (Waters Corp., Milford, MA, USA). The following acids were evaluated: acetic, citric, formic, fumaric, lactic, maleic, malic, malonic, oxalic, succinic, and quinic acids. The mushroom extracts were dissolved in Mili-Q water (Millipore, Temecula, CA, USA). Separation was conducted using Waters Atlantis C18 column (250 mm × 4.6 mm × 5 μm) at a 220-nm wavelength. The following column conditions were used: mM KH_2_PO_4_ (adjusted to pH 2.5 with H_3_PO_4_) and MeOH as an eluent (95:5, *v*/*v*), and flow rate at 0.8 mL/min [41]. Studied acids were identified by the retention times of their peaks in a chromatogram and quantified by comparing the peak area with standards (Supelco Analytical, Bellefonte, PA, USA) at a known compound concentration, according to the corresponding standard curve.

#### 2.4.7. Ergosterol Concentration

Ergosterol concentration in mushroom extracts was evaluated by UPLC (ACQUITY UPLC H-Class System and a PDA eλ Detector, Waters Corp., Milford, MA, USA) as previously described [42]. Extracts were mixed with MeOH and 2 M NaOH, irradiated twice in a microwave (2 × 15 s), cooled, and mixed with 1 M HCl. The extraction was performed with pentane and the samples were evaporated to dryness in a nitrogen stream. The ACQUITY UPLC HSS T3 C18 column (150 mm × 2.1 mm, particle size of 1.8 μm) (Waters, Dublin, Ireland) protected with a 1.7 m ACQUITY UPLC BEH C18 VanGuard Pre-column was used in the identification. The injection volume was 2 µL. The flow rate of the isocratic elution of the mobile phase (mixture of MeOH, acetonitrile, and water, 85:10:5, *v*/*v*/*v*) was 0.5 mL/min. The run time of analysis was 10 min.

#### 2.4.8. Macro and Trace Element Concentration

Extract samples were thawed and 1.00 mL of each was digested with nitric acid (3 mL) in closed Teflon vessels with the use of a Mars 6 microwave sample digestion system (CEM, Matthews, NC, USA). The two-step digestion process was performed: ramping to temperature 180 °C for 20 min and holding at 180 °C for 30 min. Following the digestion, the solution was diluted to a final volume of 5.00 mL with MilliQ water. The concentration of macro elements (Ca, Mg, K, Na, and P) and trace elements (Co, Cr, Cu, Mn, Mo, Fe, and Zn) was determined using the inductively coupled plasma optical emission spectrometer (Agilent 5110 ICP-OES, Agilent, Lexington, MA, USA) with parameters, wavelengths, analytical standards, and reference materials employed as described previously [43]. Se concentration was determined using electrothermal atomic absorption spectrometry (ETAAS) with a Zeeman background correction. Analyses were performed using the SpectrAA 280Z (Agilent Technologies, Mulgrave, Australia) instrument with pyrolytic graphite tubes and an Se hollow cathode lamp (wavelength 196.0 nm, current 10 mA, slit 1.0 nm) [44]. As a chemical modifier, palladium solution (10 μL of 500 mg L^−1^ for 20 μL of sample) was used. The limit of detection was 0.01 mg kg^−1^ and the uncertainty of results were obtained at the 5% level.

### 2.5. Statistical Analyses

The results were analyzed using STATISTICA 13.0 (StatSoft, Tulsa, OK, USA). The differences in platelet and coagulation parameters between control/ASA and mushroom extracts were evaluated with the Wilcoxon signed-rank test. The relationships between the results of the ADP and ASPI tests, and between platelet aggregation/coagulation parameters and extract characteristics were determined with Spearman’s correlation coefficient (Rs). A value of *p* < 0.05 was considered to be statistically significant.

## 3. Results

### 3.1. In vitro Studies

#### 3.1.1. Aggregation Assays

**Figure 1:**
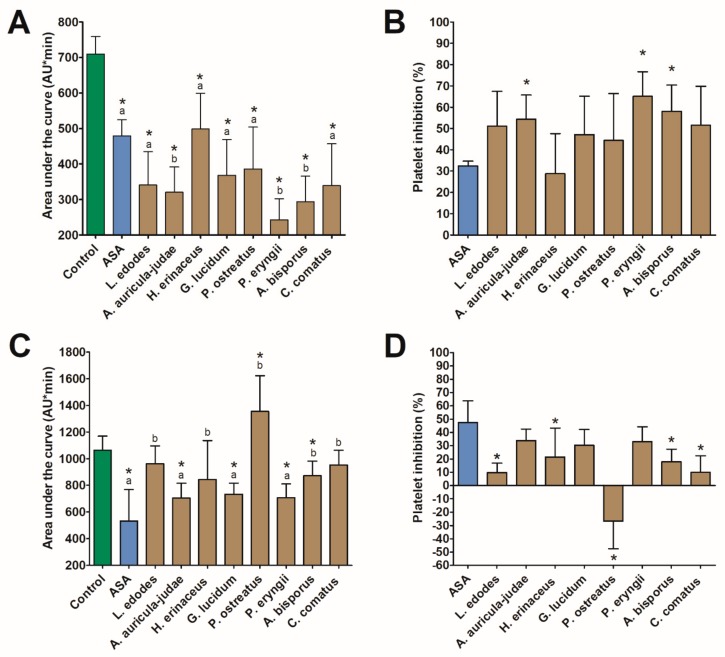
The effect (mean ± SD) of mushroom extracts on platelet aggregation (*n* = 5) induced by 6.5 µM adenosine-5′-diphosphate (**A**,**B**) and 0.5 mM arachidonic acid (**C**,**D**). The asterisk in graph (**A**), (**C**) indicates a statistically significant difference with the control while different letters denote a significant difference between the mushroom extract and 140 µmol/L of acetylsalicylic acid (ASA) (Wilcoxon signed-rank test, *p* < 0.05). The asterisk in graph B and D indicates a statistically significant difference with ASA (Wilcoxon signed-rank test, *p* < 0.05).

All investigated extracts revealed anti-aggregation activity in vitro by inhibiting platelet aggregation induced by ADP via a P2Y12 receptor. The highest platelet inhibition was observed for *P. eryngii* (by 65.1%), *A. bisporus* (by 58.0%), *A. auricula-judae* (by 54.3%), and *C. comatus* (by 51.6%) (Figure 1). In all four cases, the responses exceeded what was induced by 140 µmol/L of ASA. Extracts of the first three mushrooms and *G. lucidum* also had a significant inhibitory effect on AA-induced platelet aggregation. The highest inhibition was found for *A. auricula-judae* (by 34.0%), *P. eryngii* (by 33.1%), and *G. lucidum* (by 30.1%). In all cases, the response did not differ from that evoked by ASA. In turn, extract of *P. ostreatus* revealed an additive effect in the AA assay and increased platelet aggregation by 26.8% (Figure 1). No correlations were found between inhibition of ADP-induced and AA-induced platelet aggregation, except for the *P. eryngii* extract for which an inverse relationship was identified (Rs = −0.90, *p* < 0.05). This section may be divided by subheadings. It should provide a concise and precise description of the experimental results, their interpretation, and the experimental conclusions that can be drawn.

#### 3.1.2. Coagulation Assay

**Figure 2:**
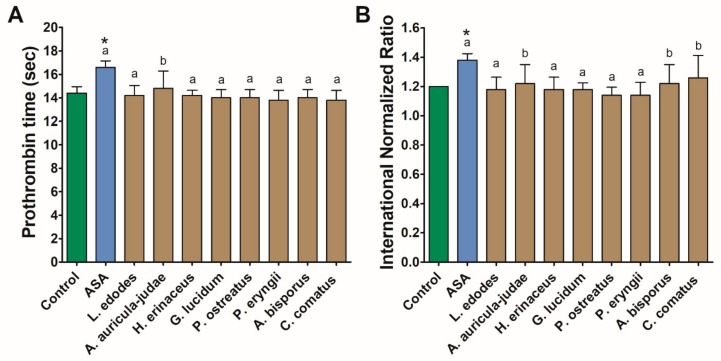
Coagulation parameters: prothrombin time (**A**) and INR (**B**) measured in human plasma treated with acetylsalicylic acid (ASA) and mushroom extracts. Asterisks indicate a statistically significant difference with the control while different letters denote a significant difference between the mushroom extract and 140 µmol/L of ASA (Wilcoxon signed-rank test, *p* < 0.05).

Treatment of human plasma with mushroom extracts did not result in altered clotting. In all cases, prothrombin time and INR values fall within the reference range and varied from 13.8 to 14.8 s, 84.2% to 89.2%, and from 1.14 to 1.26, respectively. In no case did the observed values vary significantly from the control. The highest mean values of both parameters were found for ASA and did not differ significantly from those observed in *A. auricula-judae* (prothrombin time and INR), *A. bisporus*, and *C. comatus* (INR) (Figure 2).

#### 3.1.3. Cytotoxicity and ESR Assay

**Figure 3:**
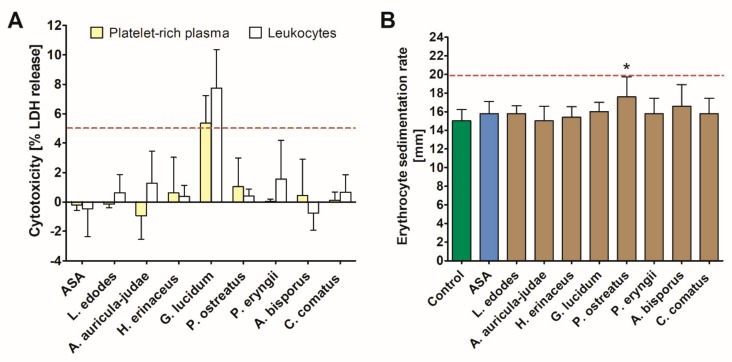
Coagulation parameters: prothrombin time (**A**) and International Normalized Ratio (INR) (**B**) measured in human plasma treated with acetylsalicylic acid (ASA) and mushroom extracts. Asterisk indicates a statistically significant difference with the control (Wilcoxon signed-rank test, *p* < 0.05).

The exposure of human PRP and leukocytes to mushroom extracts resulted in increased cytotoxicity only in the case of the *G. lucidum* extract, which caused a 5.4 ± 3.9 and 7.7 ± 4.6% LDH release, respectively. None of the investigated extracts elevated the erythrocyte sedimentation rate above a reference range of 20 mm/h. However, in comparison with the control, a significant increase in this parameter was observed in response to the *P. ostreatus* extract (Figure 3).

### 3.2. Extract Characteristics

The extract characteristics are summarized in Table 1. The highest total antioxidant capacity (TAC) and DPPH scavenging capacity were found for *P. eryngii*, *A. bisporus*, and *A. auricularia-judae*. The latter was also found to contain the highest total phenolic content. Only a few phenolic acids were identified in selected extracts: gallic in *G. lucidum* (23.7 ng/mL), trans-cinnamic in *G. lucidum* (0.06 ng/mL), and *L. edodes* (8.6 ng/mL), and 4-hydroxybenzoic in *H. erinaceus* (128.1 ng/mL). An extract of *H. erinaceum* revealed the highest concentrations of the investigated low-molecular weight organic acids: acetic, citric, fumaric, and mallic. In other extracts, the concentrations were low or undetected. The highest concentration of ergosterol was decidedly revealed by a *P. eryngii* extract followed by an extract of *L. edodes*. The highest values of total polysaccharide concentration were observed in the case of *P. eryngii*, *C. comatus*, and *A. auricularia-judae*. The main sugar components in all polysaccharide preparations were hexoses: mannose (Man), glucose (Glc), and galactose (Gal) (Table 2). The amount of Glc varied from 43% of polysaccharidic extract of *A. auricular-judae* to 83.7% in *C. comatus* and it was the main sugar component in all preparations. Small amounts of 6-deoxy-hexose (6-D-Hex), glucosamine (GlcN), xylose (Xyl), and ribose (Rib) were detected. 3-O-Methyl-hexose (3-OMe-Hex) was present (6.9%) only in the polysaccharide isolated from *A. bisporus*.

Linkage analysis showed that hexoses were mainly →4)-linked (in *P. ostreatus*, *P. eryngii*, and *C. comatus*) and →3)-linked (in *L. edodes*), whereas in polysaccharides isolated from *H. erinaceus*, *G. lucidum* and *A. bisporus* mostly branched →3,4)-linked hexose was present (Table 3). In turn, the polysaccharide isolated from *A. auricular-judae* contained mainly terminal hexose (Man)–27.57%, →3)-linked hexose (22.86%) and →3,6)-linked hexose (28.45%). It is worth noting that, in all polysaccharide preparations, a tri-branched hexose was present [→2,4,6)-linked hexose], whereas in *P. ostreatus* and *A. bisporus* a →3,4,6)-linked hexose was present, and, in *L. edodes* and *A. auricular-judae* polysaccharides, the presence of →2,3,6)-linked hexose was observed. Moreover, the polysaccharide isolated from *L. edodes* contained a multi-branched, →2,3,4,6)-linked hexose. 6-Deoxy-hexose was exclusively terminal, glucosamine was→3)-linked, whereas pentose could be either terminal, →2)-linked, or branched, i.e., →2,4)-linked.

A summary of elemental analysis is presented in Table 4. K and Fe were, respectively, the most abundant of the macro elements and trace elements in the studied mushroom extracts. A detailed elemental composition of all investigated extracts is summarized in Table 4.

**Figure 4:**
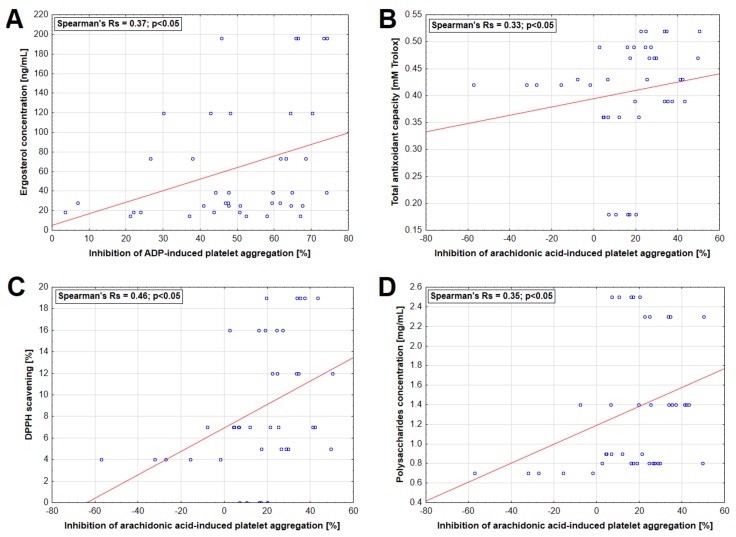
Relationship between selected characteristics of mushroom extracts and inhibition of platelet aggregation induced by 6.5 µM adenosine-5′-diphosphate (ADP) (**A**) and 0.5 mM arachidonic acid (**B**–**D**).

**Figure 5:**
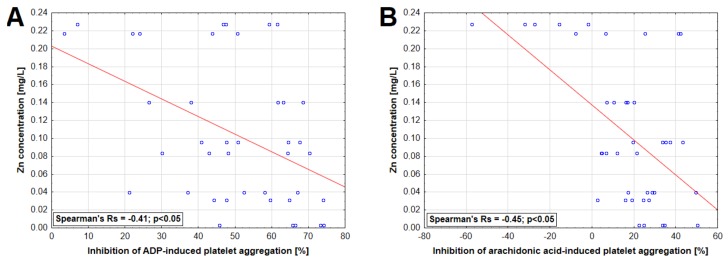
Relationship between zinc concentration in mushroom extracts and inhibition of platelet aggregation induced by 6.5 µM adenosine-5′-diphosphate (ADP) (**A**) and 0.5 mM arachidonic acid (**B**).

A statistically significant correlation was found between ergosterol concentration in extract and inhibition of ADP-induced platelet aggregation as well as between TAC, DPPH-scavenging capacity, polysaccharide concentration, and AA-induced platelet aggregation (Figure 4). Moreover, statistically significant inverse correlations were observed between Zn concentrations and the inhibition of platelet aggregation induced by ADP and AA (Figure 5).

## 4. Discussion

This study provides an overview of the potential effects of hot water mushroom extracts on the parameters of human platelet aggregation and the blood coagulation assay coupled with their chemical characterization. The intention of this research was to conduct an in vitro screening of the potential of mushrooms as functional foods for the prevention of CVD and/or cardiovascular events. For this reason, some of the most often cultivated mushroom species were employed since they are increasingly consumed in the human population in the form of various dishes and food supplements [45,46]. Moreover, the use of hot water extracts was selected since it more closely reflects the situation in which individuals consume mushrooms than extracts obtained with organic solvents. It also allows for the extraction of polysaccharides, which are considered as the major bioactive compounds of mushrooms, responsible for potential antioxidative and immunomodulating effects [47]. Selected polysaccharides isolated from mushrooms and plants have already been shown to possess anticoagulation and antiplatelet activities [48,49]. Hot water extracts are also relatively easy to prepare, which provides an additional advantage for their use on a daily basis. The results reported in the present study point the way to a preliminary selection of mushroom species whose consumption (or ingestion of how-water extracts) may have some clinical effect in primary or secondary prevention of CVD.

The most promising effects were obtained for *A. auricular-judae*, *P. eryngii*, and *A. bisporus*, which provoked the greatest inhibition of the platelet response to adenosine diphosphate and AA but did not reveal cytotoxicity. To the best of our knowledge, this is the first time that such an antiplatelet effect has been reported for *P. eryngii*, which is a species that is currently experiencing increased commercial interest but is less known than *P. ostreatus* [50,51]. Previous studies have, however, shown that another species belonging to the *Pleurotus* genus, *P. florida*, also exhibits antiplatelet activity by inhibiting adenosine 5′-diphosphate [17]. Considering that the hot water *P. ostreatus* extract investigated in the present research also evoked inhibition by the same pathway, it can be hypothesized that it is a general feature of mushrooms of this genus, related to some common constituent present in their fruiting bodies. Nevertheless, one should note that the extract of *P. ostreatus* potentiated AA-induced platelet aggregation, which is an unfavorable effect. Such an outcome was not, however, observed in the case of *P. eryngii*, which indicates some significant interspecies differences in this respect.

Generally, extracts of all the tested mushroom species investigated in the present study revealed some antiplatelet effect as shown by values obtained in ADP and ASPI tests, with the exception of *P. ostreatus* in the latter. Most importantly, however, hot water extracts of *A. auricula-judae* and *P. eryngii* not only exhibited the strong inhibition of the platelet P2Y12 ADP receptor but also inhibited the AA pathway in a manner similar to 140 µmol/L of ASA. This is a relatively valuable finding if one considers that there is clear evidence that dual antiplatelet treatment with ASA and P2Y12 receptor inhibitors is essential in the pharmacological treatment of acute coronary syndromes since it reduces the risk of ischaemic events and improves patient outcome [52,53]. The present study shows that hot water extracts of *A. auricula-judae* and *P. eryngii* may potentially provide a dual control. This is a finding requiring further confirmation in in vivo experimental models and clinical trials involving human subjects consuming these mushrooms and monitoring platelet function. However, one should bear in mind that, in the case of the *P. eryngii* extract, the inhibition in ADP-induced and AA-induced platelet aggregation was negatively correlated. This may indicate the potential competitive nature of mechanisms of action via which this extract alters platelet activation, which is a finding that requires further elucidation. However, no such relationship was observed in the case of the *A. auricula-judae* extract. Interestingly, previous studies have shown that this species contains a polysaccharide fraction capable of inhibiting platelet aggregation induced not only by adenosine diphosphate but also by collagen [48,54]. It appears that *A. auricular-judae* constituent(s) may have multifaceted pathways for decreasing platelet activities.

As observed in this study, a dual anti-platelet effect was also exhibited by an extract of *G. lucidum*. However, it also revealed cytotoxicity in platelets as well as leukocytes as shown by an increase in LDH leakage. *G. lucidum*, which is commonly referred to as Lingzhi or Reishi, is gaining more attention as a functional food [55,56]. The high content of triterpenes causes these mushrooms to taste bitter, which is why the fruiting bodies, mycelia, and spores are mostly used to produce extracts, tea, powders, or food supplements [57]. One controlled human interventional trial demonstrated that an oral dose of powdered *G. lucidum* may exert a favorable effect on the lipid profile. It highlights some potential cardioprotective activity. Nevertheless, if one considers that it did not reveal any significant effect on blood pressure and could also exert a toxic effect on platelets and other cellular blood components, its use in CVD prevention can be excluded. Yet, this by no means discredits the immunomodulatory and potential anticancer activities of polysaccharides such as the water-soluble 1,3-β-and 1,6-β-glucans isolated from *G. lucidum* [58,59]. The cytotoxic effect observed in the present study should be attributed to the triterpenoids, which are broadly represented in *G. lucidum* fruiting bodies. Selected compounds isolated from this mushroom were found to exhibit significant cytotoxic activities in vitro reported in selected cancer cell lines such as CA46, HeLa, and HL-60 [60,61]. To the best of our knowledge, this is the first report of any toxicity of constituents present in a hot water extract of *G. lucidum* in non-cancer human cells.

It is known that various agents exhibiting antiplatelet activities can also be responsible for certain coagulopathies. For example, it has been established that ASA administration can lead to prolonged prothrombin time, which results in an increased risk of bleeding [62]. This has also been observed in the present study after in vitro treatment with 140 µmol/L of ASA. Importantly, none of the studied hot water extracts affected coagulation parameters, which provides a potential additional advantage of their use as a part of prophylaxis of CVD.

Besides elucidating the effects of mushrooms on platelet function and coagulation parameters in an in vitro experimental model, the present study also provided a broad characterization of the investigated extracts. As clearly shown, the concentration of various compounds including polysaccharides, ergosterol, organic acids, and phenols as well as antioxidative activities varied significantly between certain extracts. The polysaccharides represented their main organic constituents while the concentration of phenolic compounds, organic acids, and ergosterol was low, which is due to the nature of extract preparation. As already mentioned, the polysaccharide fraction isolated from *A. auricula-judae* has already been shown experimentally to exhibit antiplatelet activity [48,54]. The present study also found a positive correlation between the concentration of polysaccharides and the inhibition of AA-induced platelet aggregation, which further justifies more in-depth investigations of the antiplatelet activities of these compounds.

Although hot water extracts cannot lead to the release of significant amounts of low-molecular weight organic acids and phenolic compounds, which are the most potent antioxidant components of mushrooms [63], the studied extracts revealed antioxidative properties as shown in TAC and DPPH assays. The highest potency in this regard was observed for the *P. eryngii* extract. This species has been previously documented to contain polysaccharides, which exhibit significant antioxidative action in vivo [64]. Importantly, the antioxidant capacity of the studied mushroom extracts remained in a positive association with the rate at which they diminished the AA-induced aggregation of platelets. It is now well established that reactive oxygen species play a significant role in the platelet activation cascade and that antioxidants such as resveratrol and Trolox can inhibit platelet responses to various stimulating events [20,65]. As shown previously, the hot water extracts of *P. eryngii* cultivated on substrates enriched with selected trace elements reveal increased antioxidative activities in isolated human platelets by preventing ROS formation and lipid peroxidation [19], which opens an interesting arena in which to test whether such an approach may also potentiate the anti-aggregative properties of this mushroom.

Interestingly, the ergosterol concentration of hot water extracts investigated in the present study also revealed a positive relationship with their antiplatelet activities in the ADP-mediated pathway. Ergosterol is a mushroom-derived vitamin D precursor since it can be transformed under UV radiation into an ergocalciferol, which is, however, less biologically active than cholecalciferol [66,67]. Calcitriol, which is an active form of vitamin D, has been shown to strongly diminish platelet aggregation in response to collagen and ADP [68]. The direct effect of ergosterol on platelet function has not received much attention so far. However, one study has shown that it can inhibit aggregation in vitro in response to various inducers [69]. Ergosterol has also been shown to partially inhibit COX-1 [70], which is an enzyme that converts AA into unstable prostaglandins, subsequently metabolized to thromboxane A2, known as a platelet activator [71]. Irreversible inhibition of COX-1 is one of the mechanisms of ASA antiplatelet action [22], even though the present study did not find any significant relationship between ergosterol concentration and the values obtained in the ASPI test. Nevertheless, it would be valuable to report the content of this sterol in any future studies by investigating the effect of mushroom consumption on platelet activity, particularly since its levels reveal interspecies and interstrain variability [38]. It would also be interesting to analyze whether supplementation of pure ergosterol would exert any anti-aggregative effects.

There was no association between concentrations of macro and trace elements in the mushroom extracts and platelet aggregation except for Zn whose concentrations revealed an inverse correlation with the platelet aggregation induced in both studied pathways. This is an interesting observation since previous studies have shown that rats fed with a high Zn diet demonstrate increased platelet responsiveness to various activators, including ADP [72]. As recently elucidated experimentally, Zn is a transmembrane agonist that induces platelet activation in a tyrosine phosphorylation-dependent manner [73]. In the present study, the highest concentration of this metal was demonstrated for the *P. ostreatus* extract, which had an additive effect on the AA-induced response. However, this observation cannot be solely explained by Zn concentration since its relatively increased concentration was also seen in extracts of *H. erinaceus* and *C. comatus*, which did not evoke such responses.

## 5. Study Limitations

Although the results of this study are interesting enough to justify further research on the antiplatelet effects of mushrooms, particularly *A. auricula-judae* and *P. eryngii*, some of the study limitations should be pointed out for a cautious interpretation. First, this study employed an in vitro experimental model and the observed effects require further confirmation, particularly in human subjects consuming the previously mentioned mushrooms. The potential group of interest includes patients with CVD or with high risk of a cardiovascular event for whom antiplatelet therapy has an established preventive role [6,7]. Therefore, the present study serves as preclinical in vitro research providing a necessary step toward testing the antiplatelet effects of mushroom extracts or cooked mushroom in patients. This could be achieved on the basis of a randomized clinical trial in which one group of patients would regularly consume mushrooms (e.g., at the usual dose of 300 g of fresh fruiting bodies per meal) for a month (or longer) while other would be given ASA as an antiplatelet agent. This study design would exclude the possibility to use a placebo. However, placebo-controlled (e.g., in cellulose) trial would be possible in case of using an encapsulated hot-water extract (or mushroom powder) administrated daily.

One should also note that the effects exerted by mushroom extracts in vitro may not be fully exerted in vivo due to potential gastrointestinal degradation of their components and limited absorption. On the other hand, the present study provides an interesting prelude for identification and isolation of particular antiplatelet agents in studied mushrooms, and investigation on the efficient method of their delivery in order to achieve a beneficial clinical outcome.

Moreover, the present study investigated the effects of hot water extracts prepared from undried fruiting bodies because species such as *A. bisporus*, *Pleurotus* sp., and *C. comatus* are mostly consumed fresh. It is not known whether their qualities would be retained after drying. It has been shown that the content of polysaccharides can decrease following such a process [74] and the extent to which it could alter the effect of the extract on platelet function remains to be investigated. As yet it is unknown whether the drying process would affect the properties reported in this case. Last but not least, the correlations between particular components of mushroom extracts and antiplatelet activities cannot be regarded as unambiguous evidence of a causal relationship but rather a suggestion to consider the content of these constituents in further research on the cardioprotective value of mushrooms.

## 6. Conclusions

The present study screened eight cultivated edible mushroom species for the potential presence of compounds affecting platelet aggregation and blood coagulation. The most promising results were obtained for *A. auricula-judae* and *P. eryngii*, which revealed a potential for dual control of platelet aggregation induced by ADP and AA. In turn, *G. lucidum* extract revealed cytotoxicity to platelets and leukocytes while the *P. ostreatus* extract had an additive effect on AA metabolism. The effect of the studied mushroom extracts on platelet function could partially be attributed to their antioxidative properties and the concentration of polysaccharides known as ergosterol and zinc. The blood coagulation parameters were not significantly altered by any mushroom extract. Further research, particularly on a clinical level, is required to confirm the cardioprotective role of *A. auricula-judae* and *P. eryngii*.

## Figures and Tables

**Table 1:** The antioxidant capacity and concentration of total phenolic compounds, organic acids, and ergosterol in the investigated, hot water extracts of mushrooms.

Parameter		Unit	*L. edodes*	*A. auricula-judae*	*H. erinaceus*	*G. lucidum*	*P. ostreatus*	*P. eryngii*	*A. bisporus*	*C. comatus*
TAC		mM Trolox equivalent	0.36	0.49	0.43	0.47	0.42	0.52	0.49	0.18
DPPH inhibition		%	7.0	19.0	7.0	5.0	4.0	12.0	16.0	0.0
TPC		µg/mL	n.d.	10.0	2.1	0.4	4.0	0.0	4.0	0.4
Organic acids	acetic	ng/mL	156.7	8.9	5380.9	104.9	n.d.	n.d.	8.8	39.3
	citric		n.d.	3.6	30.4	n.d.	n.d.	n.d.	n.d.	6.3
	fumaric		7.0	n.d.	6745.1	2.4	n.d.	n.d.	1.8	0.5
	lactic		n.d.	n.d.	n.d.	184.2	n.d.	n.d.	n.d.	15.2
	malic		n.d.	24.0	1588.0	190.3	n.d.	n.d.	n.d.	0.7
	malonic		1.6	n.d.	n.d.	n.d.	n.d.	n.d.	n.d.	n.d.
	succinic		n.d.	n.d.	n.d.	21.2	n.d.	n.d.	14.9	n.d.
	quinic		5.6	n.d.	n.d.	17.5	n.d.	n.d.	4.4	n.d.
Ergosterol		ng/mL	119.8	25.3	18.6	14.6	27.9	195.8	38.4	72.9
Total polysaccharides		mg/mL	0.92	1.41	1.38	0.79	0.67	2.30	0.78	2.50

n.d.—not detected; TAC – total antioxidant capacity

**Table 2:** The composition of sugars (µg/mg and (%) of polysaccharide fraction) in the obtained extracts of mushrooms.

Sugar Component	*L. edodes*	*A. auricula-judae*	*H. erinaceus*	*G. lucidum*	*P. ostreatus*	*P. eryngii*	*A. bisporus*	*C. comatus*
Ribose	-	trace	-	-	-	trace	3.3	-
(Rib)	-	trace	-	-	-	trace	(1.8)	-
3-*O*Me-hexose	-	-	-	-	-	-	12.3	-
(3-*O*Me-Hex)	-	-	-	-	-	-	(6.9)	-
6-deoxy-hexose	trace	7.7	7.3	19.0	8.57	trace	3.4	trace
(6-D-Hex)	trace	(2.3)	(3.5)	(2.4)	(2.8)	trace	(1.9)	trace
Xylose	trace	trace	-	-	-	trace	7.0	-
(Xyl)	trace	trace	-	-	-	trace	(3.9)	-
Mannose	107.7	145.6	12.7	138.1	32.7	29.1	17.1	trace
(Man)	(12.5)	(42.8)	(6.0)	(17.3)	(10.7)	(9.7)	(9.6)	trace
Glucose	409.9	146.4	106.5	403.5	211.7	177.4	86.0	359.7
(Glc)	(47.5)	(43.0)	(50.7)	(50.7)	(68.7)	(59.1)	(48.1)	(83.7)
Galactose	344.8	40.4	76.1	212.7	54.7	93.5	49.4	70.2
(Gal)	(40.0)	(11.9)	(36.3)	(26.7)	(17.8)	(31.2)	(27.7)	(16.3)
Glucosamine	-	-	7.3	22.8	-	trace	trace	-
(GlcN)	-	-	(3.5)	(2.9)	-	trace	trace	-

**Table 3:** The linkage structures of polysaccharides (%) isolated from investigated mushroom extracts.

Component	Retention Time [min]	*L. edodes*	*A. auricula-judae*	*H. erinaceus*	*G. lucidum*	*P. ostreatus*	*P. eryngii*	*A. bisporus*	*C. comatus*
Terminal pentose	9.24	n.d.	trace	n.d.	n.d.	n.d.	0.52	1.57	n.d.
Terminal 6-deoxy-hexose	9.90	0.33	0.44	4.73	1.94	trace	0.67	3.27	1.12
→2)-linked pentose	11.47	n.d.	n.d.	n.d.	n.d.	n.d.	n.d.	1.37	n.d.
Terminal hexose I (Man)	12.30	4.31	27.57	5.89	2.23	9.17	9.27	7.42	n.d.
Terminal hexose II (Glc)	12.37	5.16	n.d.	5.94	14.33	10.81	9.52	3.72	10.42
Terminal hexose III (Gal)	12.79	0.52	1.93	2.06	1.50	trace	0.48	2.97	1.12
→2,4)-linked pentose	14.25	n.d.	n.d.	n.d.	11.80	n.d.	3.23	2.70	n.d.
→3)-linked hexose	14.35	30.84	22.86	10.17	3.00	8.88	2.73	1.55	5.59
→4)-linked hexose	14.70	16.46	8.86	10.92	20.54	37.58	53.96	14.72	57.65
→2)-linked hexose	14.73	0.52	trace	0.54	trace	3.55	n.d.	n.d.	n.d.
→6)-linked hexose	14.94	3.51	1.47	11.45	5.37	9.20	1.21	13.74	1.78
→3,4)-linked hexose	15.63	12.33	2.62	33.61	28.10	0.56	8.46	38.05	12.22
→4,6)-linked hexose	16.75	1.37	1.47	n.d.	2.70	5.49	3.94	2.06	5.75
→3,6)-linked hexose	16.90	12.68	28.45	n.d.	1.93	2.17	0.89	1.36	n.d.
→2,4,6)-linked hexose	17.38	9.51	0.91	13.68	6.03	11.67	5.12	4.90	4.35
→3,4,6)-linked hexose	17.85	n.d.	n.d.	n.d.	n.d.	0.92	n.d.	0.61	n.d.
→2,3,6)-linked hexose	18.67	1.46	3.42	n.d.	n.d.	n.d.	n.d.	n.d.	n.d.
→2,3,4,6)-linked hexose	19.35	0.36	n.d.	n.d.	n.d.	n.d.	n.d.	n.d.	n.d.
→3)-linked hexosamine	19.95	0.64	n.d.	1.01	0.53	n.d.	n.d.	n.d.	n.d.

n.d.—not detected.

**Table 4:** The concentration (mg/L) of macro elements and trace elements in the investigated, hot water extracts of mushrooms.

Element	*L. edodes*	*A. auricula-judae*	*H. erinaceus*	*G. lucidum*	*P. ostreatus*	*P. eryngii*	*A. bisporus*	*C. comatus*
Macro Elements								
Ca	2.9	7.9	1.0	2.1	0.60	0.20	1.1	0.70
K	91.0	19.7	241	67.9	112	125	175	171
Mg	5.1	3.1	5.4	3.5	4.1	3.9	3.9	4.1
Na	2.3	2.3	0.64	0.31	1.2	0.30	2.6	3.8
P	14.5	4.6	39.3	28.2	24.8	15.2	27.5	25.5
Trace Elements								
Cr	0.073	0.018	0.018	0.014	0.014	0.069	0.009	0.009
Cu	0.013	0.017	0.14	0.002	0.019	0.044	0.074	0.15
Fe	0.77	0.26	0.67	0.083	0.20	0.22	0.10	0.24
Mn	0.11	0.017	0.11	0.029	0.040	0.030	0.029	0.036
Se	0.044	0.012	0.12	0.049	0.038	0.069	0.068	0.072
Zn	0.083	0.096	0.22	0.040	0.23	0.003	0.031	0.14
